# Impact of AYUSH 64 as an adjunctive to standard of care in mild COVID 19 - An open-label randomized controlled pilot study

**DOI:** 10.1016/j.jaim.2022.100587

**Published:** 2022-05-18

**Authors:** Anup Thakar, Mandip Goyal, Sagar Bhinde, Yagnik Chhotala, Kalpesh Panara, Swapnil Chaudhari

**Affiliations:** a Institute of Teaching and Research in Ayurveda, Jamnagar, Gujarat, India; b Department of Medicine, M P Shah Medical College, Jamnagar, Gujarat, India

**Keywords:** Ayurveda, Indian traditional system of medicine, SARS-CoV-2, AYUSH 64

## Abstract

**Background:**

Ayurveda herbal formulation AYUSH-64, proven to treat malaria and influenza-like illness in india was repurposed for COVID-19 patients considering preliminary evidances, however, scientific data was not available.

**Aim:**

To evaluate the preliminary efficacy and safety of AYUSH 64 as an add-on to standard of care in mild COVID19 patients.

**Materials and methods:**

A single centre, randomized, open-labeled, controlled, pilot study were conducted on mild COVID 19 confirmed patients. Patients allotted in interventional group (n = 41) recieved AYUSH 64, 3 gm per day in three divided dose for 14 days as an adjuvant to standard of care (SOC) whereas control group received SOC (n = 39) alone. Outcomes were reduction in WHO ordinal scale for clinical improvement, all-cause mortality, incidence of COVID19 symptoms among asymptomatic patients at baseline, use for oxygen therapy, use for a mechanical ventilator, the total duration of symptomatic phase and adverse events.

**Results:**

Mean score of WHO ordinal scale was reduced as time lapse in both the groups (f (1) = 20.5, *p* < 0.001) indicating clinical improvement among groups. There was no statistically significant difference in mean WHO ordinal scale between groups (f (1) = 0.98, *p* = 0.32). The proportion of asymptomatic patients progressing to the symptomatic stage is lower in AYUSH 64 group [OR, 0.68 (CI, 0.17–2.66)]. Mean days for the total duration of the symptomatic phase was also short in AYUSH 64 group (4.68 ± 3.29 days) compared to SOC group (5.81 ± 3.5 days). No incidence of the requirement of a mechanical ventilator, adverse drug reaction and death were observed in either of the groups.

**Conclusion:**

The findings of this pilot study show that an integrated approach of AYUSH 64 with SOC provide early trends of benefit by reduction in disease progression and in total symptom duration. However, its effects remains inconclusive on outcomes such as all cause mortality, use of oxygen therapy, invasive ventilator due to sparse outcomes.

## 1 Introduction

The Coronavirus disease 2019 (COVlD-19) pandemic had caused unparalleled public health emergency and catastrophic effect. Globally, SARS-CoV-2 has infected more than 489 million individuals which caused approximate 6.1 million deaths by April 5, 2022 [1]. Among many candidates from various categories of pharmacotherapy such as antiviral, antimalarial, steroid, monoclonal antibodies undergone scintific evaluation, only dexamethasone, remdesivir and some immunomodulatory drugs were proven to be clinically beneficial and recommended by World Health Organization (WHO) [2]. Though having vaccines and proven therapies, SARS-CoV-2 is still an issue of increasing concern with many countries enduring a second or third wave of outbreaks of this viral illness attributed mainly due to the emergence of mutant variants of the virus.

In parallel to the contemporary medicines, researches in traditional medicines are also being carried out widely to find out the solution to the pandemic. In countries such as China, Korea and India, efforts have been made to investigate the efficacy of their traditional medicines on COVID 19 [3]. Ayurveda, Yoga, Naturopathy, Unani, Siddha, and Homeopathy (abbreviated as Ayush) are five traditional therapies prevalent in India that are widely used in COVID 19. Recent research study supports the use of Ayurveda medicines as add on to conventional care in the management of mild COVID 19 [4].

Pathophysiology and clinical maping of COVID 19 considering Ayurvedic approch is possible through literature review of Ayurvdic texbooks. Patients suffering from mild COVID 19 usually have a fever and upper respiratory symptoms [5], which may be correlated with *Sannipataja Jwara* (fever dominant disease originated from vitiation of all three body humors) [6,7]. Dispite of these mild symptoms during early stage, this is to be considered as *Sannipataja* only, because of its potential to become fatal in its advanced satage.

AYUSH 64 containing four herbs namely *Kiratatikta* (*Swertia Chirata* Pexbex. Karst), *Saptaparna* (*Alstonia scholaris* R. Br.), *Kuberaksha* (*Caesalpinia bonducella* Fleming.) and *Katuki* (*Picrorhiza kurroa* Royle ex. Benth) was selected as intervention for early stage COVID 19 population considering its antipyratic properties [8,9]. An earlier clinical studies on AYUSH 64 exhibited promising results in malaria (*Vishama Jwara*) [10] and influenza-like illness (ILI) [11]. Recently, in an in-silico molecular docking evaluation of the AYUSH 64 showed that one of the ingrandients of AYUSH 64 inhibited the replication of main protease enzyme (M^pro^– Akuammicine N-Oxide) in SARS-CoV-2 [12]. ministry of Ayush, government of India also suggested use of AYUSH 64 during early stage COVID 19 [13]. Hence, the present study was aimed to evaluate the preliminary efficacy and safety of AYUSH 64 as an add-on to standard of care (SOC) in improving the clinical status of mild COVID 19 patients.

## 2 Materials and methods

### 2.1 Study site and design

A single-center, individually randomized, controlled, open-label, interventional study was conducted to assess the effectiveness of oral administration of AYUSH 64 in COVID 19 adults patients admitted to Guru Gobind Singh Hospital (GGH), Jamnagar, Gujarat, India. Site was a tertiary care Dedicated COVID 19 Hospital (DCH), level 3, run by government administration having ICU and other emergency facilities [14].

#### 2.1.1 Participants

Individuals having positive RT-PCR for SARS-CoV-2, that were admitted in the COVID ward at the GGH were screened within 48 h of their admission. Written informed consents were obtained physically from eligible participants by following COVID 19 precautions at the time of enrolment by Research Assistant.

Hospitalised mild to moderate category of COVID 19 adult patients aged 18–70 years, of either sex and having up to 4 point clinical scores as per the 9-point scale WHO ordinal scale for clinical improvement i.e Oxygen saturation ABG SPO2 atleast maintaining 94% by mask or nasal prongs were included in the study. Patients who were on a mechanical ventilator or organ support, patients unable to take oral medication, pregnant and lactating women, and patients with oncological diseases and other systemic uncontrolled conditions such as hypertension, diabetes, liver disorders, kidney malfunctions, pneumonia, acute respiratory distress syndrome, sepsis & septic shock were excluded from the study.

#### 2.1.2 Randamisation and sample size

Those who fulfilled the inclusion criteria and gave written informed consent were allocated to either of the treatment groups randomly. Random sequence was generated through computer-generated randomization software with 1:1 allocation ratio without stratification. The randomization had a block size of 18. Sample size of 80 participants was considered for the this pilot study to obtain the preliminary efficacy and safety of trial drugs.

### 2.2 Intervention

All patients, randomized to Interventional drug, received 3 g dose of AYUSH 64 per day orally in three equal divided dose at regular interval after food for 14 days as an add-on treatment to standard of care. Control group participants were given Standard of Care (SOC) following the guidelines of the Ministry of Health and Family Welfare, Government of India, which were updated from time to time, were followed by the study site for diagnosis [15] and management [16] of COVID 19 for the study. The total study duration was 28 days with 14 days of intervention and 14 days of follow-up. Compositions of AYUSH 64 is mentioned in supplementary file. AYUSH 64 was procured from the Central Council of Research in Ayurveda Science (manufactured by Unijiles life sciences LTD, Nagpur, India).

### 2.3 Outcomes

Primary Outcome Measures: Reduction in the clinical score as determined by WHO ordinal scale (0–8 point, higher point indicates disease severity) (Supplimentary file) for clinical improvement [17] and all-cause mortality which was assessed on 0, 7th, 14th^,^ and 28th day.

Secondary Outcome Measures: The incidence of COVID 19 symptoms among asymptomatic patients at baseline, use for oxygen therapy, use for a mechanical ventilator, the total duration of symptomatic phase, and adverse events were assessed on the 0, 7th^,^ and 14th day. Laboratory investigations like hemogram, alanine transaminase (ALT), Total bilirubin, blood urea, Serum creatinine, C-reactive protein (CRP), erythrocyte sedimentation rate (ESR), Ferritin, D-dimer, neutrophil-lymphocyte ration (NLR), and IgE were carried out on 0 and 14th day.

Fever, cough, dyspnea, sore throat, nausea, bodyache, abdominal pain, vomiting, nasal discharge or blockage, chest pain, anorexia, headache, and diarrhoea were considered as COVID 19 symptoms. The onset and subside date of each symptom was noted for the calculation of the total duration of symptomatic phase.

Due to the discharge policy of the study site, patients were discharged from the hospital after 14 days initially [18]. Later on, because of changes in this policy, patients were allowed to choose home isolation if they remain afebrile for at least 3 consecutive days [19,20]. For such home-isolated patients, research assistants visited home for data collections at the end of intervention and follow up to assess the outcomes.

The possible adverse events were planned to be recorded on Case Record Form and Severity of events were categorized in to mild, moderate and severe. Further, its association to the intervention was analysed and recorded in six categories (certain, probable, possible, unlikely, unclassified, unassessable) [21].

### 2.4 Statistical methods

Data were analyzed by statistical software IBM SPSS version 27. The data on continuous variables are expressed as the mean ± standard deviation and categorical variables as numbers and percentages. Pearson's chi-square and fisher's exact test (in case of small frequencies) were used for categorical variables. Mean score of WHO ordinal scale for clinical improvement at different timeline was analysed by univariate two way ANOVA considering group and time as fixed factor. Continuous variables were analyzed by paired t-test and independent t-test for Intra and Inter-Group analysis respectively. Laboratory values that did not follow normal distribution were analyzed through non-parametric tests like the Wilcoxon test and Mann Whitney U test for intra and inter Group analysis respectively. The confidence level was set at 95%, and p values of less than 0.05 were considered significant.

### 2.5 Ethical approval

The study protocol was conducted as per the Indian Council for Medical Research (ICMR) guidelines of good clinical practice and approved by the Institutional Ethics Committee, MP Shah Government Medical College, Jamnagar with approval no. IEC/Certi/98/03/2020 dated 10-06-2020. The study was registered in CTRI before the initiation of enrolment (CTRI/2020/06/025855 registered on 13/06/2020). Interim reports of the study were submitted to the Data and Safety Monitoring Board (DSMB), New Delhi for drug safety on 7th August 2020 and 15th September 2020.

## 3 Result

**Fig. 1:**
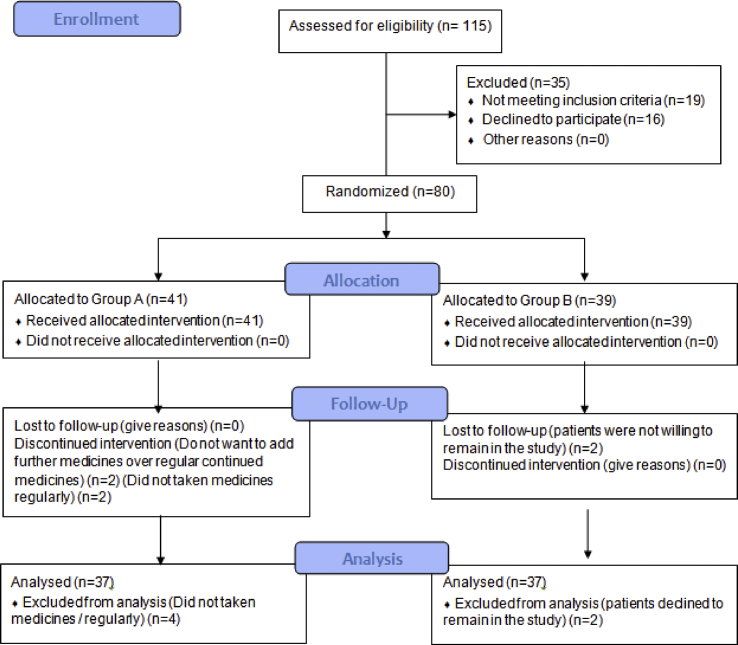
CONSORT diagram of study participants.

Total 115 COVID 19 positive patients were screened during the period of 15th June 2020 to 16th July 2020. Of these, 80 participated in the study, allocated randomly to AYUSH 64 group (n = 41) or SOC group (n = 39). From AYUSH 64 Group, 2 patients did not meet required drug compliance, and 2 refused to have AYUSH 64 in addition to ongoing standard of care after few days of enrolment. Of the 39 patients in standard of care group, two patients dropped out without giving any reasons. Hence, outcome assessment was done on 37 patients in each group (Fig. 1). Recruitment of participants in this study was stopped on 16th July 2020, as the study reached a predefined sample size for the pilot study.

### 3.1 Baseline data

Background characteristics of patients are presented in Table 1. The mean age of patients in AYUSH 64 group and standard of care group was 40 ± 12.9 and 35.31 ± 11.68 years respectively. The male: female ratio was 1.73:1 in AYUSH 64 group and 2.25:1 in Standard of care group. Sixty eight (85%) participants did not have any comorbidities while 12 (15%; AYUSH 64 group: 7; Standard of care group: 5) had comorbid conditions. Total 38 (47.5%), 24 (30%), 15 (18.75%) and 3 (3.75%) patients were having 0, 1, 2 and 3 grade on WHO ordinal scale for clinical improvement respectively.

**Table 1:** Baseline demographic and clinical characteristics.

Variables	Category	AYUSH 64 Add-on (n = 41)	Standard of care (n = 39)	*p*-value^a^
Age (Mean ± STD)	–	40 ± 12.9	35.31 ± 11.68	–
Age, categorical n (%)	18–30 years	12 (29.3%)	15 (38.5%)	0.610
	31–50 years	20 (48.8%)	18 (46.2%)	
	51–70 years	9 (22.0%)	6 (15.4%)	
Sex, n (%)	Male	26 (63.4%)	27 (69.2%)	0.582
	Female	15 (36.6%)	12 (30.8%)	
Education, n (%)	Illiterate	2 (4.9%)	4 (10.3%)	0.398
	Primary	15 (36.6%)	13 (33.3%)	
	High school	6 (14.6%)	10 (25.6%)	
	Graduate	18 (43.9%)	12 (30.8%)	
Co-morbidities n (%)	No	34 (82.9%)	34 (87.2%)	0.594
	Yes	7 (17.1%)	5 (12.8%)	
Economic status n (%)	BPL	7 (17.1%)	13 (33.3%)	0.093
	APL	34 (82.9%)	26 (66.7%)	
Marital status n (%)	Married	36 (87.8%)	30 (76.9%)	0.200
	Unmarried	5 (12.2%)	9 (23.1%)	
Habitat n (%)	Urban	33 (80.5%)	25 (64.1%)	0.184
	Semi-urban	7 (17.1%)	10 (25.6%)	
	Rural	1 (2.4%)	4 (10.3%)	
WHO Ordinal score n (%)	0	16 (39.0%)	22 (56.4%)	0.424
	1	15 (36.6%)	9 (23.1%)	
	2	8 (19.5%)	7 (17.9%)	
	3	2 (4.9%)	1 (2.6%)	
	4	0 (0%)	0 (0%)	

a Analyzed by Chi-square test; STD, Standard Deviation; n, number, %, percentage.

The data for age, sex, educational status, comorbidities, economical status, marital status, habitat and baseline status of the WHO ordinal scale was found to be distributed uniformly across both the groups (p > 0.05).

### 3.2 Outcome measures

#### 3.2.1 WHO ordinal scale for clinical improvement

**Fig. 2:**
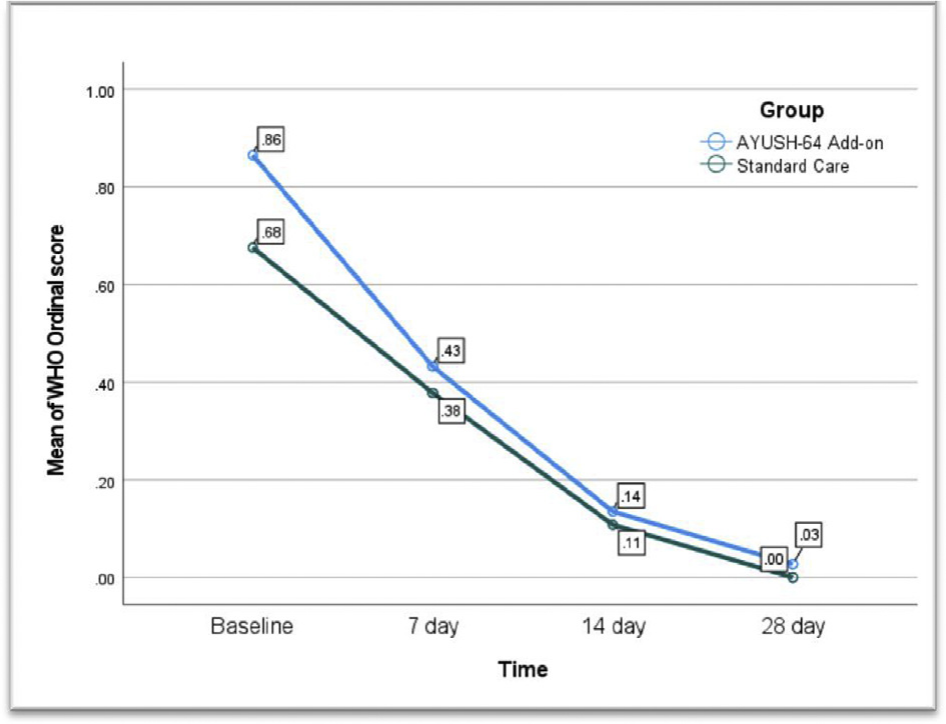
Mean score of WHO ordinal scale for clinical improvement at different point of time among the groups.

Mean score of WHO ordinal scale was reduced as time lapse in both the groups (f (1) = 20.5, *p* < 0.001) indicating clinical improvement among groups. There was no statistically significant difference in mean WHO ordinal scale between AYUSH 64 add-on and standard of care group (f (1) = 0.98, *p* = 0.32). No interection was observed between both independent variables (timeline and type of intervention) (Fig. 2).

#### 3.2.2 Incidence of COVID 19 symptoms among asymptomatic patients

At baseline, in AYUSH 64 group and standard of care group, 14 and 21 participants were found to be asymptomatic respectively. Symptoms of COVID 19 developed in 6 (42.83%) patients of AYUSH 64 group and 11 (52.38%) of Standard of care group among those asymptomatic at baseline. The percentage of asymptomatic patients progressing to the symptomatic stage is lower in AYUSH 64 group, however, the point estimate for the odds ratio was 0.68 (CI, 0.17–2.66) and statistically not significant (Table 2).

**Table 2:** Number of patients who developed COVID 19 symptoms.

Groups	Incidence of symptoms n (%)		Risk Estimate Odds ratio (CI)	*P*-value (Analyzed by Chi-square test)
	No	Yes		
AYUSH 64 add-on (n = 14)	8 (57.17%)	6 (42.83%)	0.68 (0.17–2.66)	0.581
Standard of care (n = 21)	10 (47.62%)	11 (52.38%)		

n, number of participants; CI, Confidence interval.

#### 3.2.3 Use of oxygen therapy

Two **(**5.4%) patients required oxygen therapy through nasal prong in AYUSH 64 group, wherein one (2.7%) in Standard of care group. The point estimate for the odds ratio is 2.05 (CI, 0.17–23.72) which was statistically not significant (Table 3).

**Table 3:** Number of patients who needed oxygen therapy.

Groups	Oxygen therapy used n (%)		Risk Estimate Odds ratio (CI)	*p*-value (Analyzed by Fisher's Exact Test)
	No	Yes		
AYUSH 64 add-on (n = 37)	35 (94.60%)	2 (5.40%)	2.05 (0.17–23.72)	0.556
Standard of care (n = 37)	36 (97.30%)	1 (2.70%)		

n, number of participants; CI, Confidence interval.

#### 3.2.4 Total duration of the symptomatic period

Symptomatic period was calculated in patients had symptoms of COVID 19. Mean days for the total duration of the symptomatic period for symptomatic patients was lower in AYUSH 64 group (4.68 ± 3.29 days) than in Standard of care group (5.81 ± 3.5 days). But difference was not statistically significant (p 0.221) (Table 4).

**Table 4:** Total duration of symptomatic phase.

Group (n)	Mean Days	Standard Deviation	Standard Error Mean	t	*p*-value^a^
AYUSH 64 add-on (n = 29)	4.68	3.29	0.61	−1.23	0.221
Standard of care (n = 27)	5.81	3.50	0.67		

a Analyzed through independent t-test; n, number of participants.

#### 3.2.5 Effect on laboratory parameters

AYUSH 64 when given as an add-on treatment, serum creatinine decreased significantly and hemoglobin (Hb) increased significantly. The rest of the laboratory parameters showed changes that were statistically not significant. Standard of care group provided a significant increase in laboratory values of eosinophil and Hb; whereas rest of the laboratory parameters showed changes that were statistically not significant. While comparing both the Groups for laboratory parameters, except creatinine, the difference in all parameters was statistically not significant (Table 5). The changes occurring in laboratory values in either groups were in normal range for that investigation.

**Table 5:** Effect on laboratory investigation through parametric tests.

Sr. No	Investigations	Group (n)	Mean		Intra Group (Paired t-test)		Inter Group (independent t-test)		*P*-value
			Before treatment (Day 0)	After treatment (Day 14)	Std. Deviation	*P*-value	95% CI of the Diff		
							Lower	Upper	
1	Total Leucocyte Count (/cmm)	A (n = 23)	6486.96	7669.57			−1844.43	1305.30	0.732
		B (n = 23)	6365.22	7278.26	2453.25	0.088			
2	Neutrophil (%)	A (n = 23)	57.47	59.35	13.88	0.523	−7.80	8.24	0.957
		B (n = 23)	55.64	57.74	13.11	0.452			
3	Eosinophil (%)	A (n = 23)	2.04	2.61	1.69	0.121	−0.71	1.30	0.559
		B (n = 23)	1.89	2.76	1.69	**0.023**			
4	Lymphocyte (%)	A (n = 23)	36.27	35.87	14.11	0.893	−6.89	8.60	0.825
		B (n = 23)	36.80	37.26	11.85	0.855			
5	NLR	A (n = 23)	2.02	1.77	1.74	0.502	−0.87	0.94	0.940
		B (n = 23)	1.83	1.61	1.29	0.437			
6	Creatinine (mg/dl)	A (n = 23)	0.93	0.83	0.23	**0.043**	0.001	0.25	**0.047**
		B (n = 23)	0.83	0.84	0.18	0.548			
7	Urea (mg/dl)	A (n = 21)	29.68	27.95	7.10	0.164	−1.77	5.15	0.325
		B (n = 22)	27.64	26.96	3.15	0.427			
8	Hemoglobin (gm%)	A (n = 23)	12.65	13.08	0.72	**0.001**	−0.35	0.85	0.412
		B (n = 23)	12.51	12.79	1.23	**0.004**			
9	Total Bilirubin (mg/dl)	A (n = 12)	1.15	0.56	1.29	0.095	−1.13	1.21	0.947
		B (n = 14)	1.10	0.61	1.56	0.147			

A, AYUSH 64 add-on group; B, Standard of care group; n, number; CI, Confidence interval, NLR, Neutrophil Lymphocyte Ratio.

D-dimer and ferritin were significantly decreased in AYUSH 64 group and Standard of care group at the end of intervention compared to baseline, however, the difference between groups was statistically not significant. CRP was decresed in both the groups with more decrease observed in standard of care group. At the end of intervention, change in IgE and ALT were statistically not significant between the groups. ESR was significantly decreased in AYUSH 64 group while statistically not significant in Standard of care group (Table 6).

**Table 6:** Effect on Laboratory investigation through non-parametric tests.

Investigation	Group (n)	Before Treatment (Day 0)		After Treatment (Day 14)		Intra Group (Wilcoxon)		Inter Group (Mann Whitney)	
		Mean ± SD	Median (IQR)	Mean ± SD	Median (IQR)	SE	*P*-value	SE	*P*-value
D-dimer (ng/mL)	A (n = 12)	325.75 ± 162.63	362.50 (170.25–408)	165 ± 94.6	135 (98.50–189.50)	12.74	**0.012**	14.07	1.000
	B (n = 9)	393 ± 166	344 (290–502)	179 ± 106	150 (109.50–184)	8.44	**0.015**		
CRP (mg/L)	A (n = 20)	8.16 ± 10.42	3.14 (1.56–11.97)	5.65 ± 12.02	3.15 (1.74–3.72)	26.78	0.156	36.96	0.327
	B (n = 20)	40.6 ± 69.4	6.60 (1.04–52.53)	3.51 ± 3.43	2.75 (0.75–4.13)	24.85	**0.040**		
IgE (IU/ml)	A (n = 7)	233.07 ± 296.11	63.40 (57.50–640.40)	127.13 ± 207.37	75.80 (19–87)	5.91	0.091	7.00	0.366
	B (n = 6)	401.28 ± 563.3	218.50 (27.97–1198.12)	824.67 ± 1101.8	661 (36.50–2.14.20)	4.77	0.753		
Ferritin (ng/ml)	A (n = 19)	220.71 ± 210.51	154.30 (46.90–395.40)	181.43 ± 210.54	82.80 (38.73–284.60)	24.85	**0.018**	35.59	0.336
	B (n = 20)	127.3 ± 138.7	73.71 (22.26–198.70)	93.89 ± 129.4	33.39 (9.45–120.81)	26.78	**0.033**		
ALT (IU/L)	A (n = 19)	49.08 ± 75.21	28 (16–40)	25.15 ± 13.92	22 (14–32)	22.94	0.081	32.89	0.916
	B (n = 18)	30.77 ± 18.86	19.50 (16.75–43.75)	25.5 ± 14.21	20.50 (16.50–34)	21.11	0.177		
ESR (mm/hr)	A (n = 14)	17.64 ± 7.95	17 (11.75–20)	11.5 ± 6.73	9 (7–15.25)	15.87	**0.013**	19.32	0.118
	B (n = 12)	19.75 ± 22.78	9 (7–24.50)	13.08 ± 8.18	10 (7.25–20)	12.73	0.480		

A, AYUSH 64 add-on group; B, Standard of care group; CRP, c-reactive protein; IgE, Immunoglobulin E; ALT, alanine aminotransferase; ESR, erythrocyte sedimentation rate.

#### 3.2.6 Effect on use of mechanical ventilation, adverse drug reaction and all-cause mortality

No incidence was reported for the outcomes like the number of patients who required mechanical ventilation, the number of patients reporting ADR/AE, and the incidence of all-cause mortality.

## 4 Discussion

This is the first study of its kind in Gujarat, India, to evaluate the efficacy of AYUSH 64 through a randomised controlled trial (RCT) on the WHO-recommended "ordinal score for disease severity” outcome along with others. Study suggested AYUSH 64 given as an add-on to standard of care may provide possible benefits by reducing incidence of COVID 19 symptoms among asymptomatic patients and total symptoms duration to the mild COVID 19 patients than the standard of care alone. However, present study data was not statistical significant may be due to the small sample size and trivial outcome reported in participants. Present pilot study data may facilitate the estiamation of sample size calcuclation for further full-size RCT.

A recent study revealed that AYUSH 64 when given with SOC for 30 days, hastened clinical recovery around 4 days in compare to standalone SOC [23]. Similar trend was also observed in present study. Earlier studies estimated that 20% of COVID 19 affected patients require oxygen and the death rate is 3% [22], which was contrary to our findings. In present study, the use for oxygen therapy was trivial and no mortality was observed among both the groups may be because of characteristic of study population selected i.e. mild cases of COVID 19.

No changes in liver profile, kidney profile and other blood parameters in add-on ayurveda intervention group indicates the safety of AYUSH 64. Moreover, no ADE reported in the interventional group ensuring safety of AYUSH 64 when added with standard of care. The safety of AYUSH 64 on hematological and biochemical parameters in this study which is in concordance with the findings reported in earlier studies [29,30].

Laboratory values of D-dimer and ferritin were significantly decreased in both groups without significant difference between Groups. Recent studies have revealed that there is a positive association with a rise in D-dimer value and disease severity [24]. At the same time ferritin is also a key mediator of immune dysregulation, especially extreme hyperferritinemia, via direct immune-suppressive and pro-inflammatory effects, contributing to the cytokine storm [25]. It was also noted in a previous study that serum ferritin remains within the normal range (30–400 μg/L) in patients with the non-severe disease [26] and this study also supports the same observation. Recent studies have concluded that raise in CRP is positively associated with the severity of the disease [27], In this study, it was found that baseline CRP was high with many outliers, which reaches near to the normal range (0–5 mg/L) after treatment in both the groups. ESR is a non-specific measure of inflammation and according to Ayurveda *Pitta* invariably involves the pathogenesis of inflammation (*Paka*) [28]. *Tikta Rasa* (bitter taste) predominant ingredients of AYUSH 64 might have reduced the *Pitta* vitiation to provide additional statistical significant effect in ESR in interventional group. Reduction in D-dimer, ferritin, CRP, and ESR (Table 6) in both groups indicates a reduction in the severity of disease condition explaining no need of mechanical ventilator and death among study participants.

Retention of participants to the trial was also good as only about 10% participant withdrew from the trial during study period. Furthermore, cost of AYSUH 64 is comparatively quite low and may be considered as cost-saving drug as it may hasten the clinical recovery and reduce the burden of hospitalization costs. Because of the recent surge in COVID 19 cases in India, which has greatly increased the hospital burden, a full investigation on the cost-effectiveness of AYUSH 64 is also recommended using an economic model that takes into account a variety of outcomes and other health-system-related aspects.

### 4.1 Limitations of this study

This study has some limitations such as participants were included only from single-center which may be low representativeness of the global population. Being a pilot study, the sample size was small and had limited analysis of long-term outcomes. Blinding was not possible in this study, because of operational difficulties associated with drug administration regimens, preparation of placebos, and timelines necessary to initiate experimental research during an epidemic. The result of this study is only applicable to the mild COVID 19 patients. Due to the limitations of the study site, laboratory investigations were not performed on all patients.

## 5 Conclusion

Our study provides the first evidence showing the preliminary safety, efficacy and feasibility of AYUSH 64 as add-on to standard of care for mild hospitalized COVID 19 patients. The findings of this pilot study show that an integrated approach of AYUSH 64 with standard of care provide early trends of benefit by reduction in disease progression and in total symptom duration. However, its effects remains inconclusive on outcomes such as all cause mortality, use of oxygen therapy, invasive ventilator due to sperse outcomes. The results of this study will be useful to determine sample size and to plan future RCTs.

## Source of funding

This work was supported by the Institute of Teaching and Research in Ayurveda (ITRA), Institute of National Importance (INI), Ministry of Ayush, Government of India, Jamnagar, Gujarat, India, 361008.

## Authors’ disclosure statement

The authors declare that they have no competing financial interests.

## Conflicts of interest

The authors declare that they have no conflict of interest. Prof. Bhushan Patwardhan, who is acknowledged in this submission, is also the chief editor of this journal; and does not possess conflict of interest.
