# Supplementary material for: Impact of AYUSH 64 as an adjunctive to standard of care in mild COVID 19 - An open-label randomized controlled pilot study
Source: J Ayurveda Integr Med. 2022 May 18;13(3):100587. doi: 10.1016/j.jaim.2022.100587 (PMC9114151; doi:10.1016/j.jaim.2022.100587)
Supplement: Multimedia component 1 [file mmc1.docx]

**Composition of each 500 mg tablet of AYUSH 64**

| Sr. No | Ingredients | Scientific name | Part used | Ratio |
| --- | --- | --- | --- | --- |
|  | *Katuki* | *Picrorhiza* *kurroa* Royle ex Benth. | root aqueous extract | 100 mg. |
|  | *Lata Karanja* | [*Caesalpinia* *bonduc* (L.) Roxb.](http://www.theplantlist.org/tpl1.1/record/ild-927) | seed powder | 200 mg |
|  | *Kirattikta* | *Swertia* *chirata* Buch. Ham. ex Wall. | whole-plant aqueous extract | 100 mg. |
|  | *Saptaparna* | *Alstonia* *scholaris* (L.) R. Br. | bark aqueous extract | 100 mg |

**WHO ordinal scale for clinical improvement in COVID 19**

| **Patient state** | **Description** | Score |
| --- | --- | --- |
| **Uninfected** | No clinical and virological evidence of infection | 0 |
| **Ambulatory** | No limitation of activity | 1 |
|  | Limitation of activity | 2 |
| **Hospitalized mild cases** | Hospitalized, no oxygen therapy | 3 |
|  | Oxygen by mask or nasal prongs | 4 |
| **Hospitalized severe disease** | Non-invasive ventilator or high flow oxygen | 5 |
|  | Intubation and mechanical ventilation | 6 |
|  | Ventilation + additional organ support – pressor, RRT, ECMO | 7 |
| **Dead** | Death | 8 |
